# Combined transcriptomic and proteomic analyses uncover molecular basis of heat tolerance in pakchoi (*Brassica rapa subsp*. *chinensis*)

**DOI:** 10.3389/fpls.2026.1734608

**Published:** 2026-03-11

**Authors:** Lei Wang, Fangsheng Gao, Dongqin Zhang, Chenchen Sun, Hongrong Guo, Chenghui Wang, Xuechu Du

**Affiliations:** 1 College of Ecology, Resources and Environment, Dezhou University, Dezhou, Shandong, China; 2 Shandong Degao Seed Company Limited, Dezhou, Shandong, China

**Keywords:** *Brassica rapa subsp. chinensis*, heat shock proteins, heat-tolerance, proteomic, transcriptomic

## Abstract

**Introduction:**

High temperature posed a significant abiotic stress, severely limiting plant growth and development. As a cool-loving vegetable, pakchoi (*Brassica rapa subsp. chinensis*) is highly sensitive to high temperatures, yet its molecular mechanisms underlying heat stress tolerance in pakchoi are not well explored.

**Methods:**

This study employed an integrated approach combining physiological assessments with extensive transcriptomic and proteomic profiling of leaves from a heat-tolerant pakchoi line.

**Results:**

Our physiological analyses revealed that 5 days of heat stress (39/32°C, day/night) significantly impaired plant performance, resulting in a significant reduction in plant fresh weight, chlorophyll content, and critical Chl fluorescence parameters, including Fv/Fm and PI_ABS_. Furthermore, the activities of key antioxidant enzymes, peroxidase and catalase, were significantly reduced, suggesting a reduced reliance on these antioxidant enzymes for mitigating oxidative stress in heat-tolerant varieties. Subsequent integrated transcriptomic and proteomic analysis identified 4414 differentially expressed genes (DEGs) and 506 differentially abundant proteins (DAPs) under heat stress. Functional enrichment analysis demonstrated that up-regulated DEGs/DAPs were significantly enriched in pathways essential for cellular protection and energy metabolism, including protein processing in endoplasmic reticulum, starch and sucrose metabolism, glycolysis/gluconeogenesis. Conversely, down-regulated DEGs/DAPs were mainly involved in plant hormones and signaling pathway (e.g. ABA pathway, MAPK signaling), as well as secondary metabolic processes (e.g. phenylpropanoid and flavonoid biosynthesis), suggesting a strategic reallocation of cellular resources and a shift in metabolic priorities under stress. Notably, integrated omics and protein-protein interaction (PPI) analysis highlighted the privotal role of the heat shock proteins (HSPs) in mediating heat tolerance, particularly heat shock protein 70s (HSP70s), with four HSP70s identified as hub nodes in the PPI network, three of which were involved in protein processing in the endoplasmic reticulum.

**Dissusion:**

This study not only provides novel and comprehensive insights into the multi-level physiological and adaptations of pakchoi to heat stress, but also lays a robust foundation for the development of more heat-tolerant pakchoi through targeted breeding strategies.

## 1 Introduction

Over recent decades, global temperatures have risen continuously, and extreme weather events have occurred frequently, especially in tropical and subtropical regions (Lippmann et al., 2019). The adverse effects of elevated temperature on plant growth are exacerbated by climate change, and heat stress has become one of the most critical abiotic factors constraining global agricultural output (Lobell et al., 2008; Rosenzweig et al., 2014). High temperature retard plant development, disrupt cellular processes, and may lead to plant death (Kan et al., 2023). In response to elevated temperatures, plants activate a wide range of adaptive mechanisms involving complex physiological, biochemical, and molecular regulatory networks to mitigate thermal damage. These responses include the accumulation of heat-shock proteins (HSPs) to prevent irreversible protein unfolding and aggregation (Sadura et al., 2020), and the upregulation of antioxidant defense systems—comprising enzymes such as superoxide dismutase, calatase, peroxidase (Zhang et al., 2024) and glutathione peroxidase, as well as non-enzymatic antioxidants such as ascorbic acid and glutathione (Mittler, 2002; Roach et al., 2023). Plants also reconfigure endogenous phytohormones signaling pathways, including those of abscisic acid (Huang et al., 2016), auxin (Chen et al., 2024b), jasmonates (Wang et al., 2021), to initiate downstream transcription reprogramming. Furthermore, heat stress often induces the synthesis of protective secondary metabolites, such as phenolics, terpenoids, and nitrogen-containing compounds (Salam et al., 2023). Therefore, a comprehensive understanding of these regulatory mechanisms is the foundation for breeding heat-tolerant crop varieties.

Pakchoi (*B. rapa subsp. Chinensis*), a widely cultivated leafy vegetable in China and increasingly popular worldwide. Pakchoi is a cool-season crop that thrives at 18~20°C (Yu et al., 2023). Owing to its short growth cycle, pakchoi is now grown year-round, especially in the summer and early-autumn seasons to meet rising market demands. However, as a cool-loving crop, pakchoi is highly susceptible to elevated temperatures, which severely impact its yield and quality. When pakchoi is subjected to temperatures above 28°C, its growth potential will be restricted (Gao et al., 2025). Severe heat causes leaf curling, twisting, yellowing, stem elongation, and leaf narrowing, ultimately resulting in reduced marketability. Thus, it is crucial to reveal the regulatory mechanisms underlying heat tolerance for developing heat-tolerant pakchoi cultivars. A previous study showed that a total of 1220 differentially expressed genes (DEGs) were detected and 9 KEGG pathways were significantly enriched in pakchoi under heat stress (Xu et al., 2016). Gene regulation in response to heat occurs not only at the transcriptional level (Moradpour et al., 2021), but also through complex post-transcriptional and post-translational modifications (Kan et al., 2023).

Transcriptome sequencing (RNA-Seq) has been widely used to investigate the gene expression profile under biotic or abiotic stress. In flowering Chinese cabbage (Ikram et al., 2022), functional annotation of transcriptomic data has identified 15 potential heat-tolerance genes. Similarly, Qiu et al. (2017) used transcriptomic analysis to identify key DEGs involved in salt tolerance in Chinese cabbage. However, transcriptomic data alone may not fully represent cellular processes, as discrepancies between gene expression and protein abundance often occur (Lu et al., 2022). To bridge this gap, integrated transcriptomic and proteomic approaches have been utilized to reveal regulatory networks in plants under abiotic stresses. For instance, transcriptomic and proteomic profiles were used to explore the transcriptional and post-transcriptional changes in drought-stressed tomato (Liu et al., 2023). An integrated analysis has clarified cold tolerance mechanisms in rapeseed (Mehmood et al., 2021). However, the mechanisms of heat tolerance in pakchoi remain largely unexplored. Studies of high-temperature stress on *B. rapa* have mainly focused on physiological or transcriptomic analyses (Shi et al., 2023; Zhang et al., 2022), a comprehensive molecular regulatory network underlying heat stress tolerance remain to be elucidated.

In this study, we performed an integrated transcriptomic and proteomic analysis to characterize the molecular responses of pakchoi under optimal and heat stress conditions. Based on the functional annotation of differentially expressed genes (DEGs) and differentially abundant proteins (DAPs), and correlation analysis between gene expression and protein abundance, we identified key genes and pathways associated with heat tolerance. In addition, physiological indices, including fresh weight, chlorophyll content and Chl fluorescence parameters, were assayed. Our findings provide a comprehensive understanding of the molecular and physiological adaptation of pakchoi to heat stress and may serve as a foundation for breeding heat-tolerant cultivars.

## 2 Materials and methods

### 2.1 Plant materials and heat stress treatment

Seeds of the heat-tolerant pakchoi cultivar ‘Refeng’, a widely cultivated variety in China, were bred by Degao Seed Co., Ltd. (Shandong, China). Uniformly sized seeds were germinated on moist filter paper in petri dishes and incubated in the dark at 28°C. After germination, the seeds were sown directly into 50-cell plug trays containing a growth substrate composed of peat, vermiculite, and perlite (1:1:1, v/v/v). Seedlings were cultivated in artificial climate chambers set at 25/18°C (day/night) with a 16 h/8 h light/dark photoperiod, a light intensity of 12000 lux, and a relative humidity of 70%. At the 4–5 true leaf stage, half of the seedlings were randomly assigned to the heat stress group (HS) and exposed to a high temperature regime of 39/32°C (day/night). The remaining seedlings were maintained under the original ambient conditions (25/18°C, day/night) as control group (CK). To evaluate the heat tolerance of pakchoi, leaf samples were collected following a 5-day treatment period according to the protocols of Xu et al. (2016) and Yuan et al. (2025). Specifically, the second leaf from the apex was sampled from each seedling in both HS and CK groups, immediately frozen in liquid nitrogen, and finally stored at -80°C for measurement of physiological indexes, and transcriptomic and proteomic analyses. Moreover, fresh weight of shoot and root tissues were recorded. The experiment was performed in three independent biological replicates, each comprising twenty seedlings.

### 2.2 Chlorophyll content

Chlorophyll content was assayed using the ethanol extraction method (Yu et al., 2023). Briefly, fresh leaf tissue (0.5 g) was homogenized in 96% (v/v) ethanol and centrifuged at 15,000 × *g* for 3 min. Absorbance of the supernatant was measured at 665, 649, and 470 nm, respectively, to calculate *Chla* and *Chlb*. Total chlorophyll was defined as the sum of *Chla* and *Chlb*.

### 2.3 Chl fluorescence parameters

After 5 days of heat stress treatment, fully expanded and healthy upper leaves of HS and CK were randomly selected for leaf OJIP transient measurements using a Handy Plant Efficiency Analyzer (Handy-PEA, King’s Lynn, Norfolk, UK) as described by Teng et al. (2022). Leaves were dark-adapted for at least 30 min to ensure complete closure of all photosystemII (PSII) reaction centers. Then, the measurements were initiated by applying a beam of saturating red light (peak wavelength 650 nm, intensity 3000 µmol·m^-2^·s^-1^). The resulting OJIP transients were analyzed with Biolyzer HP3 software (Bioenergetics Laboratory, University of Geneva, Switzerland) to generate energy pipeline models. The *Chl* fluorescence parameters *Fv/Fm* and *PI_ABS_* were calculated as described by Li et al. (2019).

### 2.4 Antioxidant enzyme activities

Activities of Peroxidase (POD) and Catalase (CAT) were determined using commercial kits (Solarbio Life Sciences, Beijing, China; Cat#BC0095, Cat#BC0205) in accordance with the manufacturer’s instructions (Zhang et al., 2022). Three biological replicates (*n=3*) were analyzed for each sample.

### 2.5 Transcriptome sequencing and analysis

Total RNA was isolated from leaf tissues using TRIzol Reagent (Thermo fisher scientific, USA) following the manufacturer’s protocol (Zheng et al., 2022). The concentration, purity, and integrity of the RNA were quantified using a NanoDrop 2000 (NanoDrop, USA) and 1.0% agarose gel electrophoresis. For library construction, 1 µg of total RNA per sample was used. The mRNA was isolated using oligo (dT) beads and fragmented in fragmentation buffer. First-strand cDNA was synthesized using random primers followed by second-strand synthesis and end-repair. The library was sequenced on the Illumina NovaSeq X Plus platform (Majorbio BioPharm Technology Co., Ltd., Shanghai, China). To obtain clean reads, low-quality reads and adapter sequences were removed using fastq. Then, the Q20, Q30, and GC content of the clean data were calculated. Clean reads were mapped to the assembled *Brassica rapa subsp. pekinensis* genome (http://www.bioinformaticslab.cn/EMSmutation/download/). Filtered reads were counted using RSEM (version 1.3.3). Differential expression analysis was performed using DESeq2 (version 1.38.0) (Varet et al., 2016). Functional annotation of transcripts were conducted using GO, KEGG, NR, Swiss-Prot, Pfam and EggNOG databases. Three biological replicates were performed in transcriptomic analysis.

### 2.6 RT-qPCR validation of gene expression

The cDNA was synthesized from purified RNA using the *Evo M-MLV* RT Mix Kit with gDNA Clean for qPCR (AG, Changsha, China). RT-qPCR was performed using a SYBR Green Premix Pro Taq HS qPCR Kit (AG, Changsha, China). The *BcGAPC* gene was used as an internal control (Gao et al., 2025). The sequences of all gene-specific primer are provided in **Supplementary Table 1**. Relative gene expression levels were calculated using the 2^-ΔΔCT^ method.

### 2.7 Proteome sequencing and analysis

#### 2.7.1 Protein extraction and digestion

Protein extraction and digestion were performed as previously described by Mu et al. (2024). Briefly, leaf tissue was ground in liquid nitrogen and lysed in a solution containing 8 M urea, 1% SDS, and a protease inhibitors. Samples were sonicated under cryogenic conditions for 30 min and centrifuged at 16000 × g at 8°C for 30 min. Supernatants were collected to determine protein concentration using a BCA kit (Thermo Scientific). For each sample, 100 µg of protein was reduced with 100 mmol·L^-1^ triethylammonium bicarbonate buffer (TEAB) and 10 mmol·L^-1^ Tris (2-carboxyethyl) phosphine (TCEP) at 37°C for 1 h, followed by alkylation with 40 mmol·L^-1^ iodoacetamide (IAM) in the dark at room temperature for 40 min. The proteins were then precipitated and digested overnight at 37°C with trypsin (trypsin: protein, 1:50, w/w).

#### 2.7.2 Peptide quantification, 4D-DIA (four-dimensional data-independent acquisition) mass detection and protein identification

After protein digestion, peptides were desalted using HLB cartridges, vacuum-dried, and reconstituted in 0.1% trifluoroacetic acid (TFA). Peptides concentrations were determined using ultraviolet spectrophotometry by Nano Drop One (Thermo Scientific). LC-MS/MS analysis was performed on a timsTOF Pro2 mass spectrometer (Bruker, Germany) coupled with a Vanquish Neo UHPLC system (Thermo Scientific) at Majorbio Bio-Pharm Technology Co. Ltd. (Shanghai, China). Briefly, peptides were loaded onto a reversed-phase C18 column (Ionopticks, 1.6 μm, 75 μm×250 mm) and separated using a 44-minutes linear gradient from mobile phases A (water with 2% ACN and 0.1% formic acid) to B (water with 80% ACN and 0.1% formic acid). The mass spectrometry scanning range was 100–1700 m/z. The data obtained from mass spectrometry were analyzed using Compass HyStar software (Bruker, Germany).

### 2.8 Integration analysis of transcriptome and proteome profiles

Integrated proteomic and transcriptomic analysis was conducted using the Majorbio online platform (http://www.majorbio.com/). KEGG enrichment analysis and protein-protein interaction analysis were conducted. Network visualization was carried out using Cytoscape 3.7.2.

## 3 Results

### 3.1 Effects of heat stress on morphological and physiological indexes

**Figure 1:**
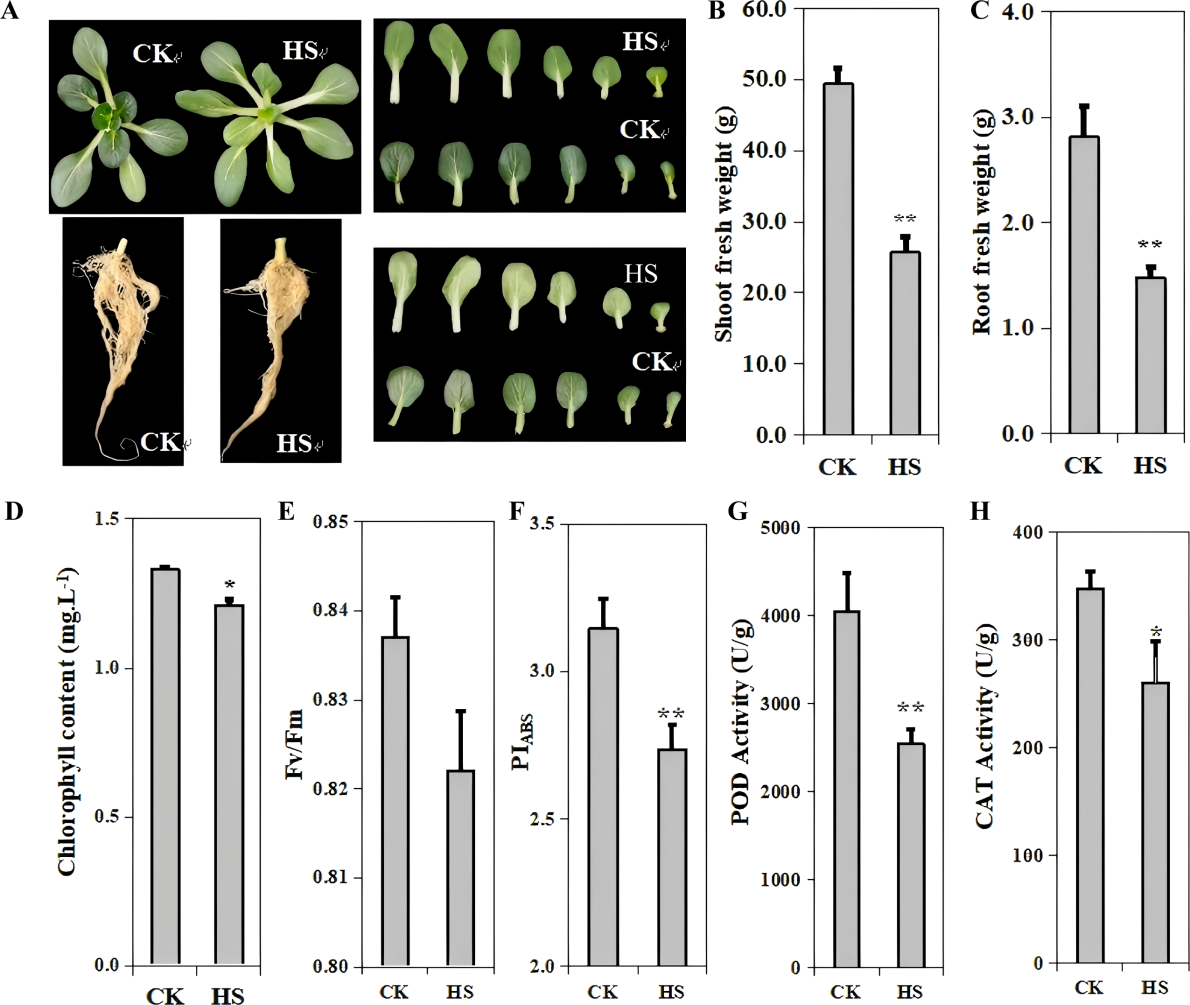
Phenotypic and physiological changes of HS and CK. **(A)** plant shoot and root changes. **(B)** Shoot fresh weight. **(C)** Root fresh weight. **(D)** Chlorophyll content in leaves after heat stress. **(E)** Maximum quantum efficiency of photosystem II, Fv/Fm. **(F)** Performance index on absorption basis, PIABS. **(G)** Peroxidase (POD) activity. **(H)** Catalse (CAT) activity. ** and * indicates a significant difference at P<0.01 and P<0.05 using two-tailed Students t-test.

Following 5 days of heat stress treatment, pakchoi seedlings displayed evident phenotypic differences compared to the control (CK). Leaves of heat-stressed plants (HS) exhibited mild outward curling, twisting, and chlorosis (**Figure 1A**). Shoot and root fresh weights were significantly reduced under heat stress compared to CK (**Figures 1B, C**). Leaf chlorophyll content in HS-treated leaves was markedly lower than that in CK plants (**Figure 1D**). Heat stress also impaired photosynthetic efficiency, with a significant reduction in chlorophyll fluorescence parameters, including Fv/Fm and PI_ABS_ (**Figures 1E, F**).

To further evaluate physiological stress responses, the activity of POD and CAT were measured. Both enzyme activities were significantly decreased in HS plants compared to CK (**Figures 1G, H**). POD and CAT activities decreased by 37.09% and 25.27%, respectively, in HS compared to CK.

### 3.2 Differentially expressed genes and functional annotation

Each library generated 40.74 to 50.61 million raw reads. Average clean reads of 44.84 and 42.03 million were obtained from HS and CK samples, respectively. All libraries showed high sequencing quality, with Q30 values above 95%, and GC content between 47.28% and 48.10% (**Supplementary Table 2**).

**Figure 2:**
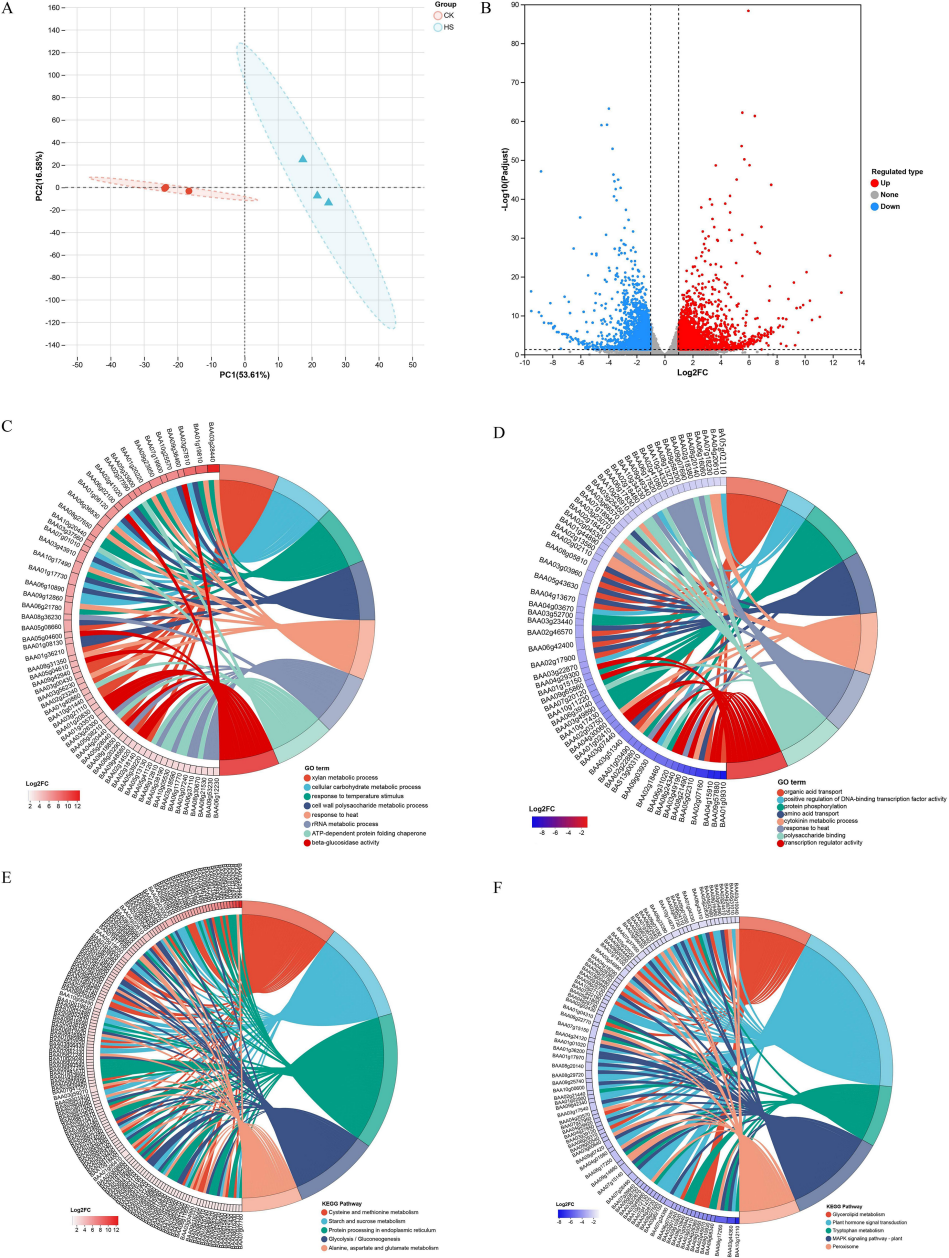
Overview of the differentially expressed genes (DEGs) in response to heat stress. **(A, B)** PCA and volcano plots showing differentially expressed genes. **(C)** Up-regulated DEGs in GO enrichment analysis. **(D)** Down-regulated DEGs in GO enrichment analysis. **(E)** Up-regulated DEGs in KEGG enrichment analysis. **(F)** Down-regulated DEGs in KEGG enrichment analysis.

Principal component analysis (PCA) showed a good cluster between biological replicates, as well as a clear separation between HS and CK group (**Figure 2A**). A total of 34,860 genes were obtained, among which 4,414 were identified as differentially expressed (|log2FC|≥1 and *P*-adjust<0.05). Of these, 2459 DEGs were up-regulated and 1955 DEGs were down-regulated in the HS group (**Figure 2B**; **Supplementary Table 3**).

GO enrichment analysis identified a total of 186 significantly enriched GO terms (*P*-adjust <0.05), including 110 biological processes, 58 molecular functions, and 18 cellular components (**Supplementary Table 4**). Up-regulated genes were significantly enriched in xylan metabolic process (GO:0010383), response to temperature stimulus (GO:0009266), response to heat (GO:0009408), ATP-dependent protein folding chaperone (GO:0140662), cellular carbohydrate metabolic process (GO:0044262), cell wall polysaccharide metabolic process (GO:0010383), rRNA metabolic process (GO:0016072), and beta-glucosidase activity (GO:0008422) (**Figure 2C**). Conversely, down-regulated genes were significantly enriched in 96 GO terms, including organic acid transport (GO:0015849), positive regulation of DNA-binding transcription factor activity (GO:0051091), protein phosphorylation (GO:0006468), amino acid transport (GO:0006865), cytokinin metabolic process (GO:0009690), response to heat (GO:0009408), polysaccharide binding (GO:0030247), and transcription regulator activity (GO:0140110) (**Figure 2D**).

KEGG enrichment analysis showed that up-regulated genes were primarily enriched in metabolism-related pathways, including cysteine and methionine metabolism (map00270), starch and sucrose metabolism (map00500), alanine, aspartate and glutamate metabolism (map00250), glycolysis/gluconeogenesis (map00010), and protein processing in endoplasmic reticulum (map04141) (**Figure 2E**). Down-regulated genes were mainly enriched in pathways related to plant hormone signal transduction (map04075), MAPK signaling (map04016), glycerolipid metabolism (map00561), tryptophan metabolism (map00380), and peroxisome (map04146) (**Figure 2F**).

### 3.3 Differentially abundant proteins and functional analysis

**Figure 3:**
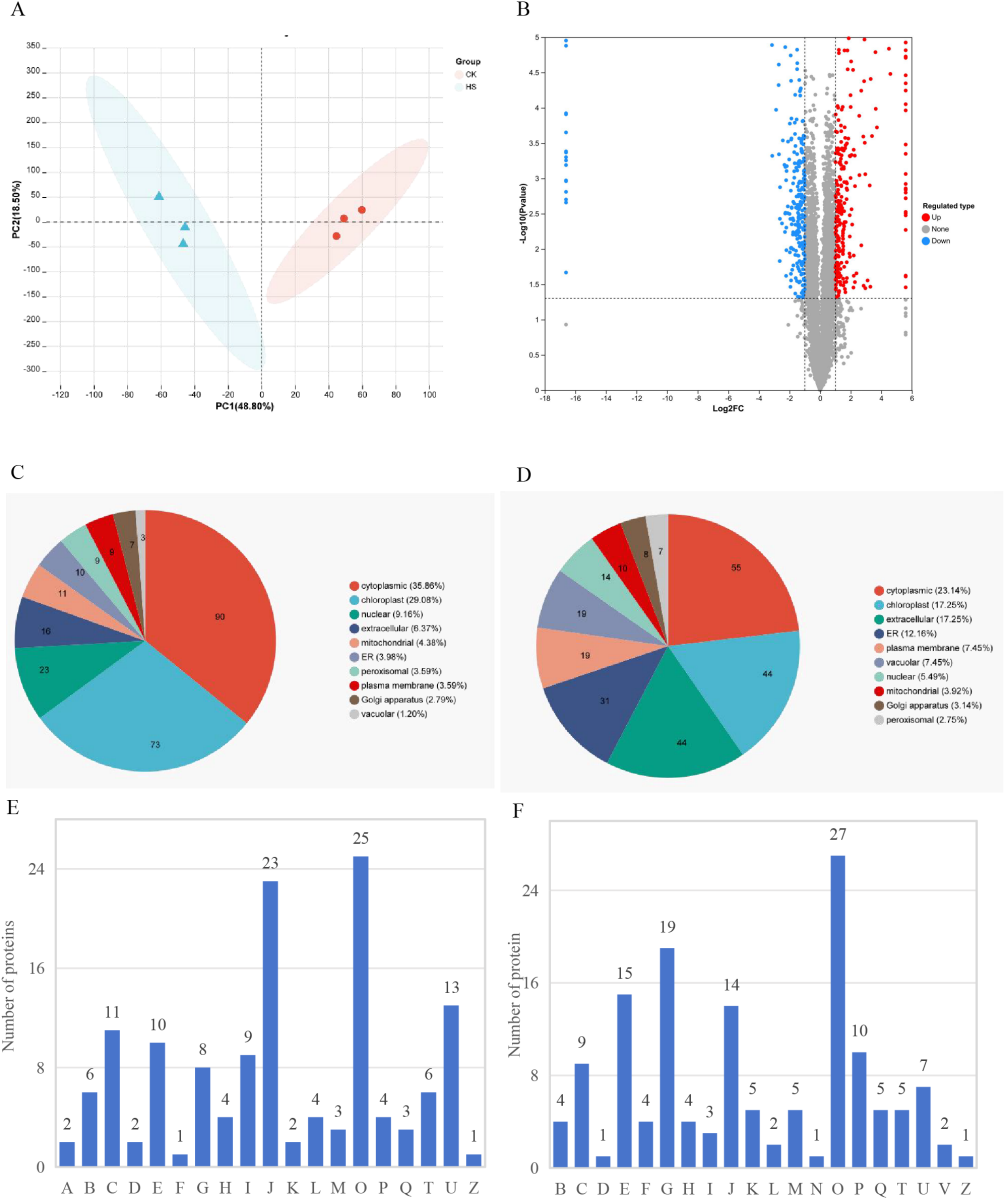
PCA **(A)** and volcano plots **(B)** showing differentially abundant proteins. Subcellular localization chart of **(C)** Up- and **(D)** Down-regulated protein. EggNOG functional classification chart of **(E)** Up- and **(F)** Down-regulated proteins.

The numbers of identified precursors, peptides and proteins in each sample were presented in **Supplementary Table 5**. The majority of identified proteins exhibited low sequence coverage, with 38.42%, 25.49%, and 27.26% of proteins in the range of 0-10%, 10-20%, and 20-40%, respectively (**Supplementary Figure 1A**). Furthermore, most proteins were covered with only one peptide (**Supplementary Figure 1B**). The molecular masses of the identified proteins were predominantly distributed in 20–60 KDa (**Supplementary Figure 1C**). PCA revealed clear separation between HS and CK, while three biological replicates within each group clustered closely, indicating high reliability of the experiment results (**Figure 3A**).

Based on established cut-off criteria (|log2FC| >1.5 (up-regulated) or < 0.667 (down-regulated) and *P* < 0.05), a total of 506 differentially abundant proteins (DAPs) were identified, of which 251 proteins (50%) showed a higher abundance and 255 proteins (50%) showed a lower abundance in the HS group compared to CK group (**Figure 3B**; **Supplementary Table 6**).

To understand the biological functions of the DAPs, we conducted subcellular localization prediction and EggNOG (Evolutionary Genealogy of Genes: Non-supervised Orthologous Groups) functional classification. The subcellular localization prediction showed that most up-regulated proteins were located in cytoplasmic (90 DAPs, 35.86%), and chloroplast (73 DAPs, 29.08%) (**Figure 3C**). The down-regulated proteins were mainly located in the cytoplasmic (55 DAPs, 23.14%), chloroplast (44 DAPs, 17.25%), extracellular (44 DAPs, 17.25%), and endoplasmic reticulum (31 DAPs, 12.16%), accounting for 69% (**Figure 3D**). DAPs were mainly related to ‘posttranslational modification, protein turnover, chaperones’ (**Figures 3E, F**).

**Figure 4:**
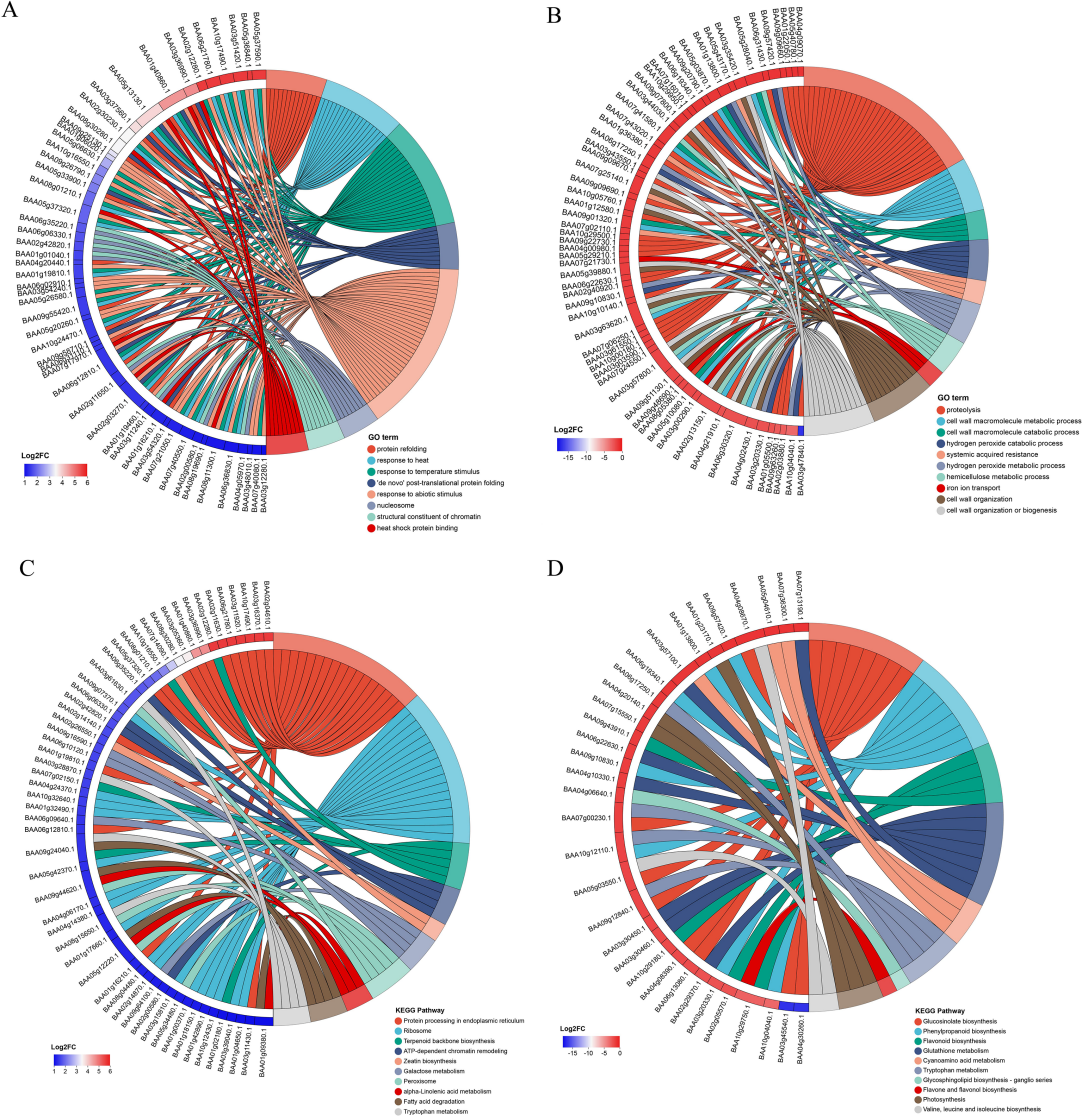
Enrichment diagram of GO and KEGG differentially abundant proteins (DAPs). **(A)** Up-regulated DAPs in GO enrichment analysis, **(B)** Down-regulated DAPs in GO enrichment analysis, **(C)** Up-regulated DAPs in KEGG enrichment analysis, **(D)** Down-regulated DAPs in KEGG enrichment analysis.

GO enrichment analysis showed that up-regulated proteins were enriched in response to heat, response to abiotic stimulus, protein refolding and folding, spermidine and polyamine metabolic process in the biological process category, heat shock protein binding, protein heterodimerization activity, protein folding chaperone in the molecular function category, and nucleosome, protein-DNA complex and central vacuole in the cellular component category (**Figure 4A**; **Supplementary Table 7**). Down-regulated proteins were mainly enriched in proteolysis, cellwall macromolecule metabolic process, hydrogen peroxide catabolic process and carbohydrate metabolic process in the biological process category, catalytic activity, peptidase-related activity, hydrolase activity, heme binding, and tetrapyrrole binding in the molecular function category, extracellular region, apoplast, external encapsulating structure in the cellular component category (**Figure 4B**; **Supplementary Table 8**).

KEGG pathway analysis showed that up-regulated proteins were significantly enriched in protein processing in endoplasmic reticulum and ribosome pathways (**Figure 4C**). Down-regulated proteins were primarily enriched in metabolism-related pathways, including glucosinolate biosynthesis, phenylpropanoid biosynthesis, flavonoid biosynthesis (**Figure 4D**).

### 3.4 Joint analysis of DEGs and DAPs

**Figure 5:**
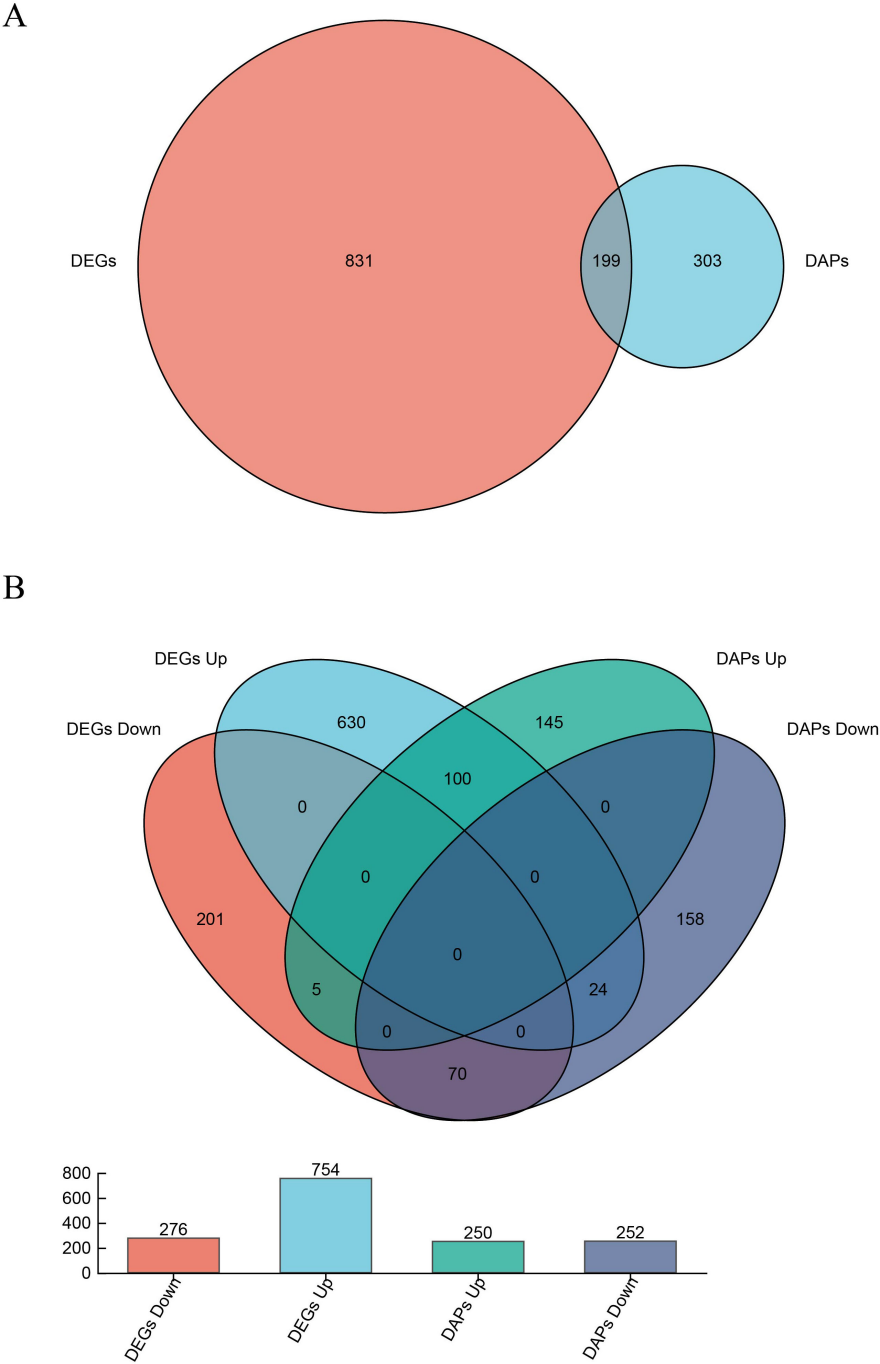
Joint Venn analysis for total DEGs/DAPs **(A)** and shared factors **(B)** in samples. (“UP” and “DOWN” represent up/down-regulated expression of genes or proteins).

The RNA-seq and proteomic data were integrated to reveal the coordinated regulation of gene expression and protein abundance under heat stress. A total of 199 genes were significantly regulated at transcription and translation levels (**Figure 5A**), of which 100 were up-regulated and 70 were down-regulated (**Figure 5B**). Interestingly, 24 genes were up-regulated at the transcript level but down-regulated at the protein level, whereas 5 showed the opposite trend (**Figure 5B**).

**Figure 6:**
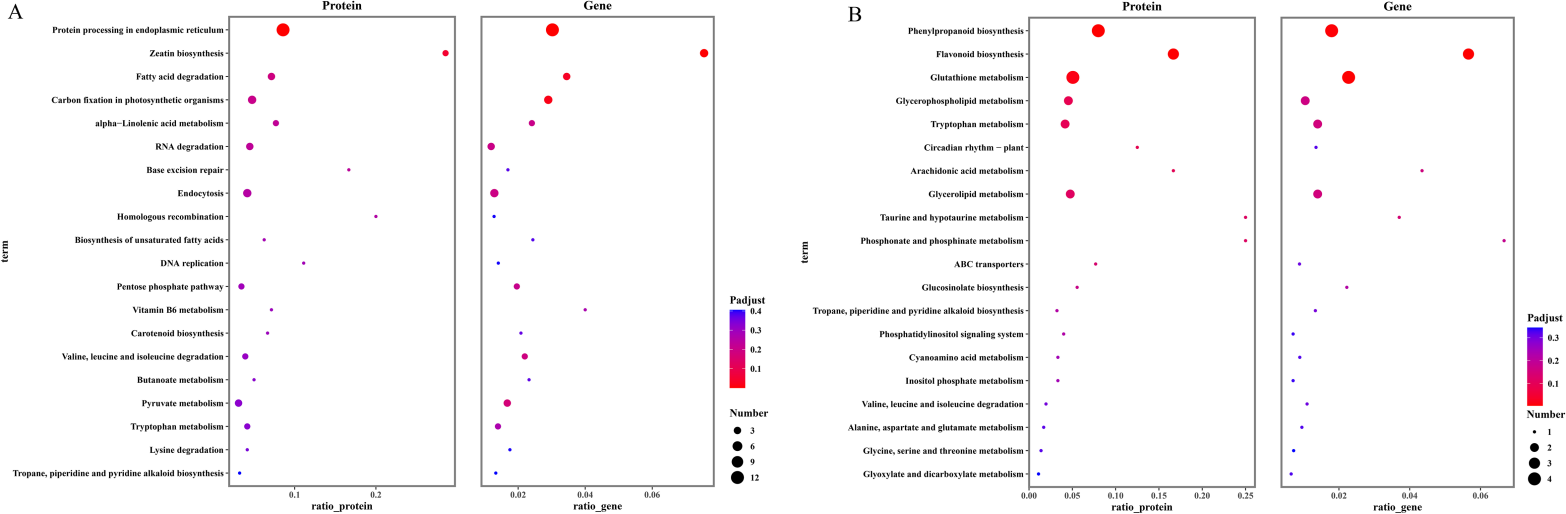
KEGG pathway enrichment analysis. **(A)**: the top 20 pathway of up-regulated DEGs/DAPs according to the enrichement degree. **(B)** the top of 20 pathway of down-regulated DEGs/DAPs according to the enrichment degree.

KEGG enrichment analysis of these co-regulated DEGs and DAPs revealed that the 100 up-regulated genes and proteins were enriched in 40 distinct pathways (**Figure 6A**). Notably, the pathway “protein processing in the endoplasmic reticulum” was the only one significantly enriched by both DEGs and DAPs, comprising 12 gene-protein pairs, including 11 heat shock proteins (HSPs). Among them were seven Hsp20s (heat shock protein 20) and four Hsp70s (heat shock protein 70). Additional pathways enriched by up-regulated DEGs alone included zeatin biosynthesis, fatty acid degradation, and carbon fixation in photosynthetic organisms (**Figure 6A**). Conversely, the 70 co-downregulated gene-protein pairs were significantly enriched in the phenylpropanoid biosynthesis and flavonoid biosynthesis (**Figure 6B**). These pathways are associated with the production of secondary metabolites that contribute to plant defense and adaptation, and their suppression suggested a reallocation of cellular resources under heat stress. Together, these integrated results emphasize the central role of heat shock proteins and metabolic reprogramming in pakchoi’s heat stress response.

### 3.5 Protein-protein interaction network analysis of cor-DEGs-DAPs

**Figure 7:**
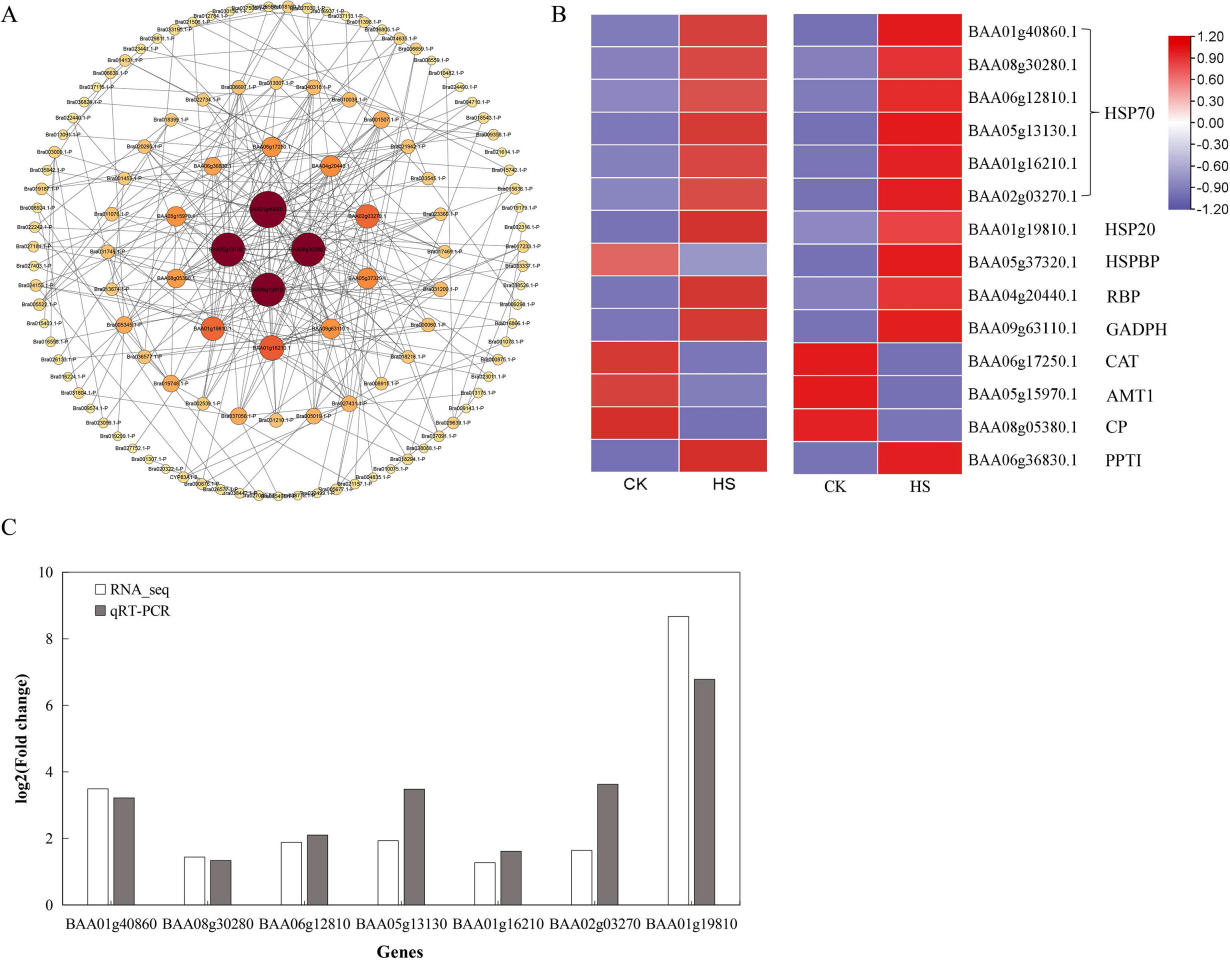
**(A)** PPI network of 199 correlated differentially expressed genes and differentially abundant proteins (cor-DEGs-DAPs). **(B)** Heat map of the 14 core protein in transcript and protein levels. **(C)** Seven DEGs were validated by comparison of RNA-Seq and RT-qPCR in leaf of HS compared with CK.

To elucidate the molecular mechanisms underlying the heat stress response, a protein-protein interaction (PPI) network was conducted using the 199 genes and proteins that were co-differentially expressed at both transcript and protein levels (cor-DEGs-DAPs). This network aimed to identify hub DEGs/DAPs and their interactive relationships involved in heat tolerance. Topological network analysis of the PPI network identified four key proteins: BAA01g40860.1, BAA08g30280.1, BAA06g12810.1, and BAA05g13130.1 (**Figures 7A, B**), all of which belong to HSP70 family and are located in the cytoplasm. Heatmap analysis confirmed that both mRNA expression and protein abundance of these HSP70 members were significantly up-regulated under heat stress. Additionally, the second layer of the PPI network comprised 10 cor-DEGs-DAPs, including two chloroplast-localized HSP70 members (BAA01g16210.1 and BAA02g03270.1), one HSP20 (BAA01g19810.1), and one HSP-binding protein (BAA05g37320.1). These findings collectively highlight six HSP70s, one HSP20 and one HSP binding protein formed a core interacting complex in the network, and the critical role of HSP70 located in cytoplasmic and chloroplast in mediating heat tolerance in pakchoi. Notably, BAA01g40860.1, BAA08g30280.1, BAA06g12810.1 and BAA01g16210 were found to be grouped in a significant KEGG pathway of protein processing in endoplasmic reticulum. Genes corresponding to these core HSPs were validated by real-time RT-PCR, and the results were consistent with RNA-seq data (**Figure 7C**).

The hub protein BAA01g40860.1(HSP70) was directly linked to 27 cor-DEGs-DAPs, including 17 HSPs, which mediate protein folding and degradation of misfolded proteins, 5 photosynthesis-related enzymes, including BAA04g20440.1 (rubisco large subunit-binding protein, RBP), BAA09g63110.1 (glyceraldehyde-3-phosphate dehydrogenase, GAPDH), BAA05g06630.1 (Ribulose bisphosphate carboxylase/oxygenase activase, RCA), BAA05g14370.1 (Oxygen-evolving enhancer protein, OEE), BAA09g35060.1 (thioredoxin, Trx), 4 antioxidant enzymes, including BAA03g30460.1 (glutathione S-transferase, GST), BAA02g29370.1 (Glutathione S-transferase, GST), BAA03g30450.1 (Glutathione S-transferase, GST) and BAA06g17250.1 (Catalase, CAT). Interestingly, BAA01g40860.1 (HSP70) also interacted with *BAA09g07340.1*, a gene encoding a histone deacetylase, suggesting that epigenetic regulation might also contribute to the heat stress response in pakchoi.

**Figure 8:**
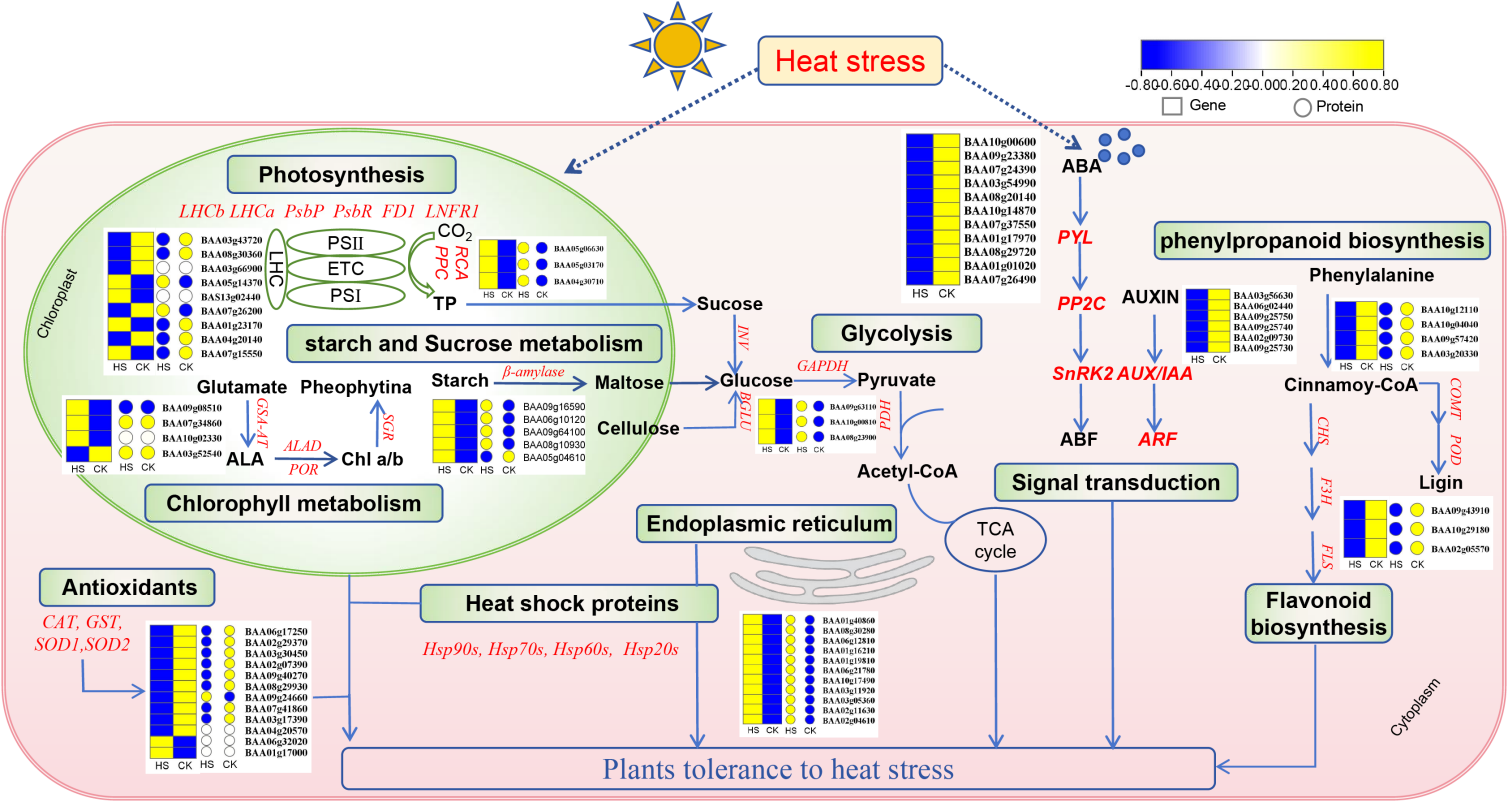
A schematic model summarizing the response to heat stress in pakchoi. Heat maps was generated using log2(FC) of mRNA (box) and protein (circle) levels in HS and CK.

To summarize the transcriptional and translational responses of pakchoi under heat stress, a schematic model was constructed integrating key genes and proteins identified in the multi-omics analysis (**Figure 8**). Heatmaps based on log_2_ fold-change values were used to visualize expression trends at both the mRNA (box) and protein (circle) levels. Under heat stress, major alterations occurred in photosynthesis, plant signal transduction, antioxidant systems, endoplasmic reticulum, starch and sucrose metabolism, and secondary metabolite biosynthesis. For example, the expression of genes encoding light-harvesting complex proteins (*LHCb*, BAA03g43720, BAA08g30360), *LHCa* (BAA03g66900) were significantly down-regulated at the mRNA or protein level compared to CK. In contrast, PsbP-encoding genes (BAA05g14370, BAS13g02440) at mRNA level and PsbR (BAA07g26200) at protein level were significantly up-regulated under heat stress. Moreover, the abundance of ferredoxin (BAA01g23170, BAA04g20140, FD1) and ferredoxin-NADP^+^ reductase (BAA07g15550, LNFR1) were all down-regulated in response to heat stress (**Figure 8**).

## 4 Discussion

As a cool-season vegetable, pakchoi is particularly vulnerable to heat stress. Under temperature above 28°C, plant growth and development are inhibited, ultimately impairing yield and quality (Gao et al., 2025). In this study, shoot and root fresh weight of pakchoi were significantly lower than that of CK after 5 days of heat treatment with 39/32°C (day/night). Compared with CK, the leaves had mildly outward curling, twisting and chlorosis, which had already exhibited preliminary heat damage symptoms. Leaf chlorophyll content under HS was significantly lower than that of CK. Heat-induced leaf chlorosis is a common symptom observed in diverse plant species, including rice (Abdelaal et al., 2021), tomato (Zhou et al., 2017), sorghum (Gosavi et al., 2014), and wheat (Hu et al., 2020; Roy et al., 2021). However, the underlying mechanisms during heat stress remain elusive. Whether heat-induced chlorophyll loss is caused by heat-induced inhibition of chlorophyll synthesis and/or heat-enhanced chlorophyll degradation in plant leaves. We found that the expression of genes encoding glutamate-1-semialdehyde aminotransferase (BAA09g08510, GST-AT), delta-aminolevulinic acid dehydratase (BAA07g34860, ALAD) and protochlorophyllide reductase (BAA10g02330, POR) favorable for chlorophyll synthesis, were significantly up-regulated, and the expression of genes encoding magnesium dechelatase (BAA03g52540, SGR) favorable for chlorophyll-degradation were significantly down-regulated, whereas these genes were not significantly changed at protein level (**Figure 8**). It was likely due to a compensatory attempt by the plant in response to heat-stress, especially for heat-tolerant lines. Previous studies in Bentgrass have reported that the chlorophyll-synthesizing enzyme did not differ significantly after heat stress, but the activites of chlorophyll-degrading enzyme, including chlorophyllase and peroxidase increased significantly (Rossi et al., 2017). The discrepancy may be attributed to variations in stress intensity and the specific physiological responses of different plant species. Moreover, Chlorophyll biosynthesis may be inhibited by high temperature-induced protein denaturation (Park et al., 2007), substrate limitation (Barry, 2009), or structural damage to chloroplasts (Dutta et al., 2009).

It is well documented that high temperature can cause direct damage to the photosynthetic apparatus (Mathur et al., 2014). The chlorophyll fluorescence parameters Fv/Fm and PI_ABS_ are highly sensitive to such damage, as indicators for evaluating plant photosynthetic performance and their health status under stress (Diao et al., 2014). A value of Fv/Fm close to 0.83 is widely regarded as an indicator of plant healthy state (Li et al., 2019). In this investigation, the Fv/Fm of pakchoi seedlings with HS treatment is close to 0.81, indicating that the plant has a certain tolerance to heat stress, which confirmed that the selected cultivars in this trial are heat-tolerant varieties. The PI_ABS_ dropped greatly under stress, which was much more sensitive than the Fv/Fm, which is consistent with the previous discovery (Li et al., 2019; Zushi et al., 2012). KEGG enrichment analysis showed that DEGs and DAPs were also enriched in Photosynthesis - antenna proteins (map00196) and photosynthesis pathway (map00195). Chlorophyll a/b binding proteins are vital components of the light-harvesting complex (LHC), and are also known as photosynthesis-antenna proteins (Liang et al., 2008). In the present study, genes encoding LHCb and LHCa were significantly down-regulated at the mRNA or protein level compared to CK. This finding contrasts with the previously reported induction of 34 *GaLHC* genes in pepper under abiotic stress (Shuo et al., 2024), suggesting that the regulation of light-harvesting complexes may be dependent on stress conditions.

In higher plants, PS II extrinsic proteins, including PsbO, PsbP, PsbQ, and PsbR, are essential for maintaining oxygen evolution under physiological conditions (Sasi et al., 2018). We observed significant up-regulation of *PsbP* at the mRNA level and increased abundance of *PsbR* at protein level, implying that their dominant roles in regulating and stabilizing PS II under heat stress, as also supported by earlier work (Kimura et al., 2002). A similar finding was reported in sweet potato, where up-regulation of *IbPsbP* and its interaction with *IbOr* enhanced PSII efficiency during heat stress (Kang et al., 2017). Studies in *Arabidopsis* have also shown that PsbR was required for maintaining its conformation and stabilizing PsbP and PsbQ binding in PS II (Allahverdiyeva et al., 2007). Moreover, the abundance of *FD1* and *LNFR1* were all down-regulated under heat stress, which were consistent with observations in cold-stressed *Brassica napus* (Mehmood et al., 2021). Within the carbon fixation process, rubisco activase, encoded by BAA05g06630, was up-regulated at both mRNA and protein level (**Figure 8**). Given that rubisco activase thermostability is a critical factor in plant heat tolerance (Qu et al., 2023), its induction may represent an adaptive strategy in pakchoi. Similarly, two phosphoenolpyruvate carboxylase (PEPC) genes (BAA04g30710 and BAA05g03170) were significantly up-regulated at protein level (**Figure 8**), which may help alleviate the metabolic loss of the C3 pathway due to heat stress (Bayramov, 2017; Doubnerová and Ryšlavá, 2011).

KEGG pathway analysis demonstrated that the up-regulated DEGs were primarily enriched in metabolism-related pathways, including cysteine and methionine metabolism (map00270), starch and sucrose metabolism (map00500), alanine, aspartate and glutamate metabolism (map00250), glycolysis/gluconeogenesis (map00010). Among these, starch and sucrose metabolism, and glycolysis/gluconeogenesis have been widely reported to play important roles in plant response to abiotic stress (Li et al., 2024; Xing et al., 2021). In the starch and sucrose metabolism pathway, 33 up-regulated DEGs were identified, encoding β-glucosidase, α-glucosidase, β-fructofuranosidase, β-amylase, endoglucanse and hexokinase. This finding aligns with previous reports in red raspberry (Guo et al., 2023a) and *Pinus massoniana* (Li et al., 2024), which also observed the enrichment of these enzymatic genes under heat stress. Moreover, proteomic analysis revealed that only β-fructofuranosidase (BAA09g16590, BAA06g10120, BAA09g64100) and β-amylase (BAA08g10930) were consistently up-regulated at the protein levels, while β-glucosidase (BAA05g04610) was down-regulated (**Figure 8**). Previous studies have proved that glucose acts as both a signaling molecule and a metabolite involved in osmotic regulation and quick energy supply (Sami et al., 2016). Thus, we propose that activities of β-amylase, β-fructofuranosidase, and β-glucosidase (Majoul et al., 2004) contribute to keep glucose homeostasis under heat stress, thereby conferring protective effects to enhance plant heat-tolerance (Chen et al., 2024a). In the glycolysis/gluconeogenesis pathway, up-regulation of DEGs and DAPs encoding pyruvate dehydrogenase (BAA10g00810, BAA08g23900) and Glyceraldehyde-3-phosphate dehydrogenase (BAA09g63110) (**Figure 8**), promoted synthesis of pyruvate and acety-CoA to sustain plant energy metabolism under heat stress (Rollins et al., 2013).

Joint two-omics KEGG enrichment analysis revealed that both down-regulated DEGs and DAPs were highly enriched in ‘phenylpropanoid biosynthesis’ and ‘flavonoid biosynthesis’ pathways. In phenylpropanoid biosynthesis, four DEGs/DAPs, including *COMT* (BAA10g12110) encoding flavone O-methyltransferase and three peroxidase genes (*POD*: BAA10g04040, BAA09g57420, BAA03g20330), showed decreased expression under HS compared to CK (**Figure 8**). These enzymes contribute to lignin biosynthesis, and the down-regulation of *POD* observed here is consistent with results reported in tea plants under high temperature (Zheng et al., 2024). Peroxidase catalyze the final step of the lignin biosynthesis (Rao and Barros, 2024; Vanholme et al., 2019), and the reduced transcript and protein abundance of POD align with the lower peroxidase activity detected in heat-stressed leaves.

In the flavonoid biosynthesis pathways, several key enzymes such as chalcone synthase (CHS), chalcone isomerase (CHI), flavonol synthase (FLS), flavanone 3-hydroxylase (F3H), flavonoid 3’-monooxygenase (CYP75B1), dihydroflavonol 4-reductase (DFR), and flavonoid 3-O-glucosyltransferase (UFGT) (Pandey et al., 2016), are known to regulate flavonoid metabolism under abiotic stress, including drought (Ma et al., 2014), salt (Pi et al., 2018), and heat (Guo et al., 2023b). In this study, genes encoding F3H (BAA09g43910), FLS (BAA10g29180) and CHS (BAA02g05570*)* were down-regulated at both mRNA and protein level under heat stress (**Figure 8**). A similar down-regulation of flavonoid-related genes, such as *CHI*, *HCT*, *FLS*, and *DFR* in *Rosa hybrida* (Wang et al., 2024), and *HCT* and *DFR* in *Pinellia ternata* (Guo et al., 2023b) have been reported under heat stress. These results suggest that heat stress leads to down-regulation of key flavonoid biosynthesis genes, which the specific genes may vary with different crops.

Plant hormones and MAPK signaling pathways play important roles in mediating plant adaptation to abiotic stress (Qin et al., 2022; Wang et al., 2024). In this study, down-regulated DEGs were enriched in ‘Plant hormone signal transduction’ and ‘MAPK signaling pathway’, indicating their coordinated involvement in heat adaptation (Kotak et al., 2007). In particular, multiple DEGs associated with the abscisic acid (ABA) pathway were suppressed under heat stress, including genes encoding ABA receptors (PYL: BAA10g00600, BAA09g23380, BAA07g24390), protein phosphatases (PP2C: BAA03g54990, BAA08g20140, BAA10g14870, BAA07g37550, BAA01g17970 and BAA08g29720) and protein kinases (SnRK2: BAA01g01020, BAA07g26490) (**Figure 8**). These data indicated that ABA, a well-known ‘stress hormone’, participates in heat stress response in pakchoi (Danquah et al., 2014). Typically, under abiotic stress conditions, ABA binding to PYL receptor inhibits PP2C phosphatase activity, thereby releasing SnRK2 kinases to phosphorylate downstream targets to initiate stress-responsive genes expression. Previous studies have reported that *PYL* and *PP2C* were up-regulated to enhance plant resistance to abiotic stress (Nian et al., 2021; Wang et al., 2012), whereas *PYL* and *PP2C* were down-regulated under heat stress in our study. It may be attributed to a negative-feedback regulatory mechanism: ABA accumulation over time during stress suppress downstream genes *PYL* and *PP2C* expression (Di et al., 2018; Merlot et al., 2001). Auxin has been reported as a negative regulator in plant stress resistance (Kazan, 2013). Studies have found that Aux/IAA proteins significantly inhibited auxin-signal transduction and might improve resistance to heat stress (Chen et al., 2024b). We also observed *Aux/IAA* (BAA03g56630, BAA06g02440) *and ARF* (BAA09g25750, BAA09g25740, BAA02g09730, BAA09g25730) were significantly down-regulated to enhance heat resistance in pakchoi (**Figure 8**).

Heat shock proteins (HSPs) are an important class of molecular chaperones (Park and Seo, 2015) that play an essential role in maintaining cellular homeostasis in response to environmental stresses (Jacob et al., 2017; Waters and Vierling, 2020), especially heat stress (Grigorova et al., 2011). In the present study, we identified twenty HSPs and chaperones induced by heat stress, including nine HSP20s, seven HSP70s, two HSP60s, one HSP90 and one HSP-binding protein. Interestingly, PPI network analysis using a total of 199 cor-DEGs-DAPs revealed that six HSP70s, one HSP20 and one HSP-binding protein constituted a core interacting complex, with four HSP70s (BAA01g40860.1, BAA08g30280.1, BAA06g12810.1 and BAA05g13130.1) acting as hub nodes in the network (**Figure 8**). These findings supported previous studies and highlighted the central and pivotal role of the heat shock proteins (HSPs) in mediating heat tolerance (Grigorova et al., 2011; Sadura et al., 2020), particularly HSP70s. Previous research also showed that HSP70s have a better protective effect as crucial markers and response proteins to heat stress in *Zostera japonica* (Chen and Qiu, 2022)*, Rosa hybrida* (Wang et al., 2024), and *Nicotiana tabacum* (Zhang et al., 2023).

Integrated transcriptomic and proteomic KEGG enrichment analysis revealed that most HSPs, including three core protein HSP70s (BAA01g40860.1, BAA08g30280.1 and BAA06g12810.1) were significantly enriched in ‘protein processing in the endoplasmic reticulum’ (**Figure 8**). The endoplasmic reticulum (ER), as the large intracellular organelle, is essential for the translation, translocation, glycosylation and folding of cell membrane and secreted protein (Gao et al., 2023). Under heat stress, the demand for protein processing often exceeds the folding capacity of the ER, resulting in ER stress (Singh et al., 2021). These enriched HSPs likely help to mitigate ER stress by facilitating the refolding or clearance of misfolded proteins. For example, the HSP70 (*BAA01g40860.1*) enriched in the ER protein processing pathway was linked with some key enzymes involved in photosynthesis, and antioxidant enzymes, which maintain key metabolism pathway under heat stress.

Enhancing antioxidant defenses is another strategy for alleviating heat-induced oxidative damage. We found the majority of the *CAT* family (BAA06g17250) and *GST* family (BAA02g29370, BAA03g30450, BAA02g07390, BAA09g40270, BAA08g29930, BAA09g24660, BAA07g41860 and BAA03g17390) DEGs/DAPs were down-regulated in response to heat (**Figure 8**), which was consistent with the reduced CAT activity measured in heat-stressed leaves. Similar suppression of antioxidant enzymes was found in other crops (Lu et al., 2008; Zhao et al., 2022), suggesting a reduced dependence on enzymatic antioxidant systems in heat-tolerant varieties, possibly due to a greater reliance on HSPs for protection. The previous study on Chinese cabbage indicated a negative correlation between SOD activity and heat tolerance (Shi et al., 2023). No significant change in protein abundance levels of SOD may be related to heat-resistance of the variety used in this experiment (**Figure 8**). Besides for these enzymes, sugars as the new emerging reactive oxygen species scavengers, are able to participate in plant defense responses, which needs to be verified in future studies.

## 5 Conclusions

In summary, our study investigated the physiological indicators and revealed dynamic expression changes of genes/proteins in response to heat stress. Heat stress led to a significant decrease in plant fresh weight, chlorophyll content, and Chl fluorescence parameters, including Fv/Fm and PI_ABS_. Transcriptomic and proteomic analysis identified 4414 differentially expressed genes and 506 differentially abundant proteins under heat stress. The significant up-regulated DEGs/DAPs were found to be enriched in protein processing in endoplasmic reticulum, starch and sucrose metabolism and glycolysis, whereas the significant down-regulated DEGs/DAPs were found to be enriched in ‘Plant hormone signal transduction’, ‘MAPK signaling pathway’, ‘phenylpropanoid biosynthesis’ and ‘flavonoid biosynthesis’. Joint two-omics and PPI analysis demonstrated that seven HSP70s, one HSP20 and one HSP binding protein formed a core interacting complex in the regulatory network, and four HSP70s acting as the hub node, which highlighted the central role of the heat shock protein family, particularly HSP70s, in mediating heat tolerance. Our results offer valuable insights into the molecular mechanisms of the heat tolerance in pakchoi.

## Data Availability

The transcriptomic data were deposited in the China National Center for Bioinformation database, under accession no. CRA029725 for the publicly accessible site http://ngdc.cncb.ac.cn/gsa. The mass spectrometry proteomics data have been deposited to the ProteomeXchange Consortium via the PRIDE partner repository with the dataset identifier PXD068141, https://www.ebi.ac.uk/pride/archive/projects/PXD068141.
