# Supplementary material for: Combined transcriptomic and proteomic analyses uncover molecular basis of heat tolerance in pakchoi (Brassica rapa subsp. chinensis)
Source: Front Plant Sci. 2026 Mar 11;17:1734608. doi: 10.3389/fpls.2026.1734608 (PMC13014383; doi:10.3389/fpls.2026.1734608)

Figure S1A Distribution of Protein's Sequences Coverage

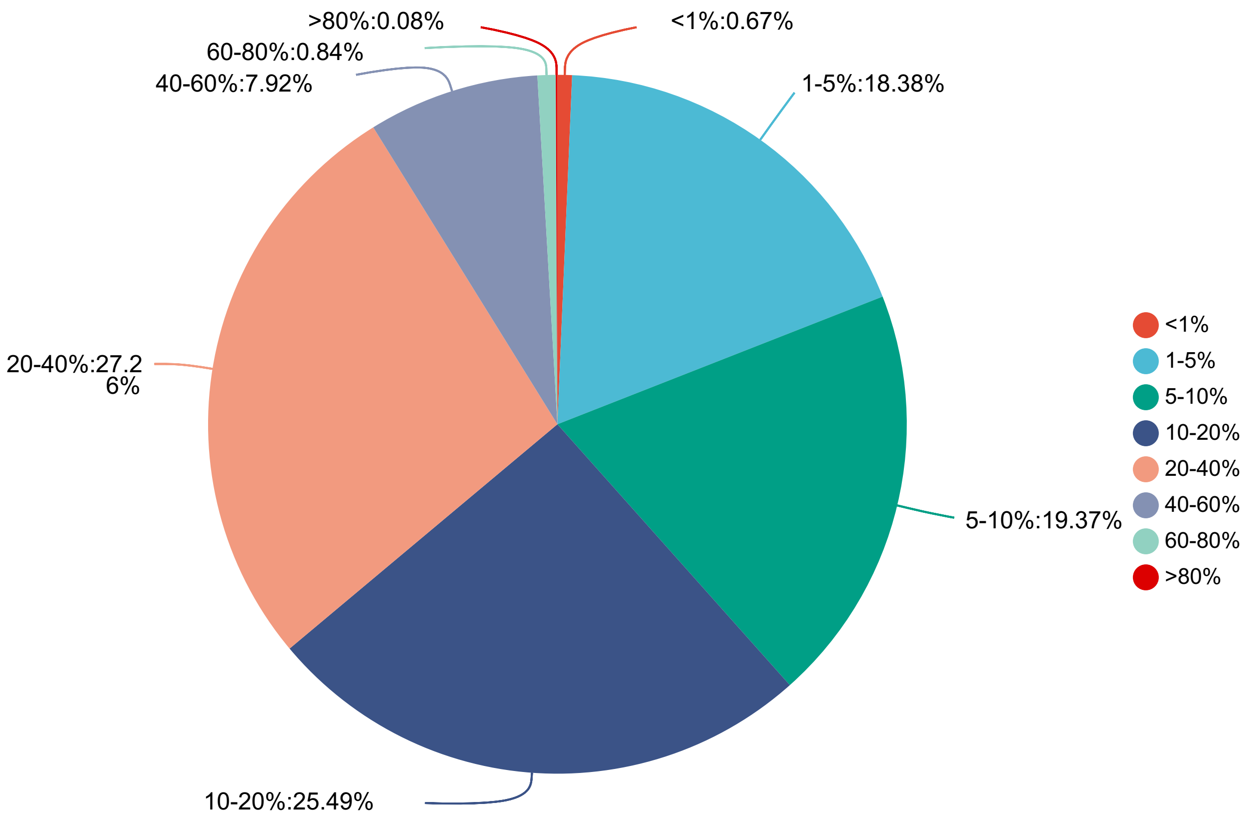

Figure S1B Peptide number distribution

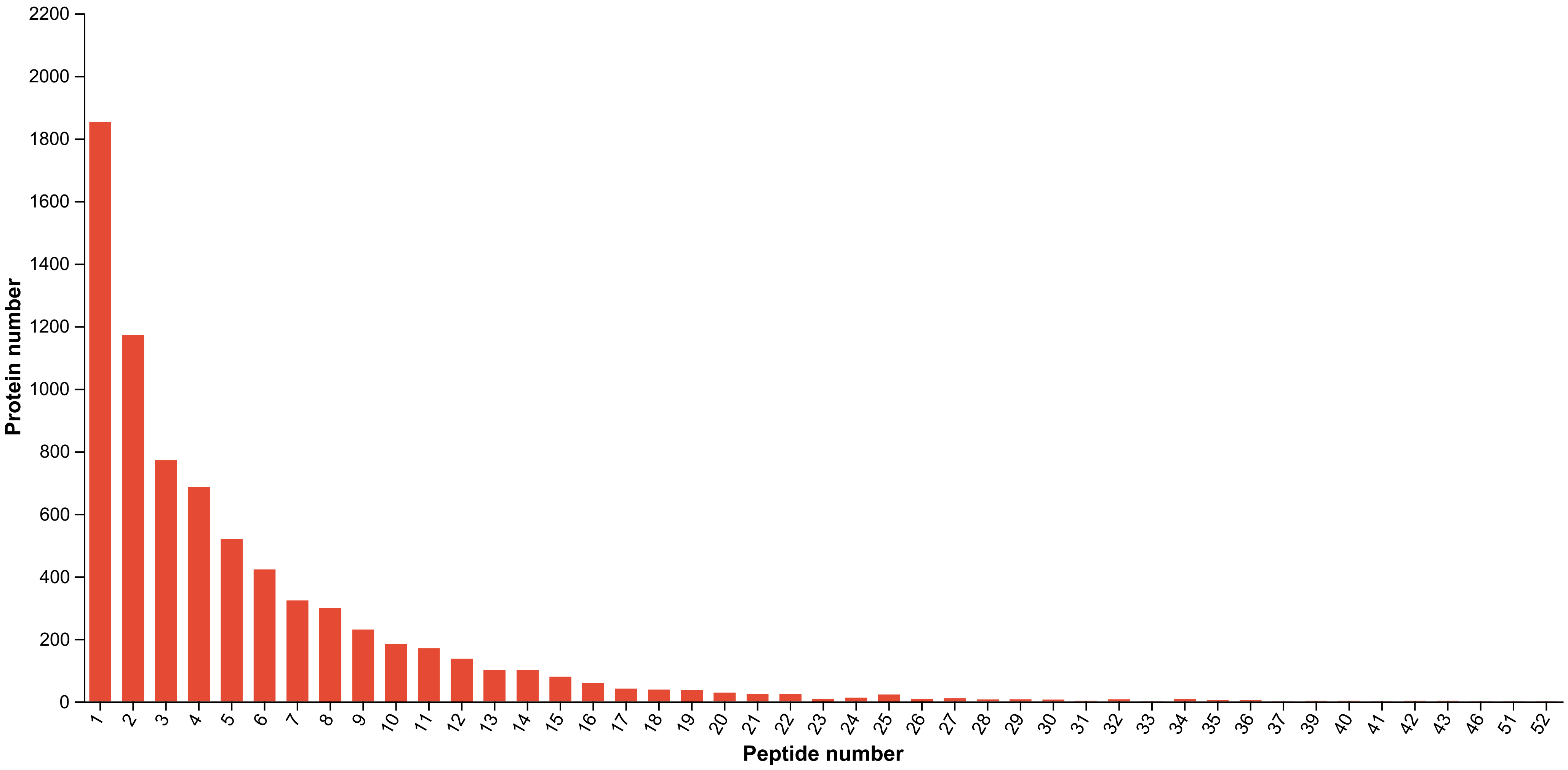

Figure S1C Protein molecular weight distribution

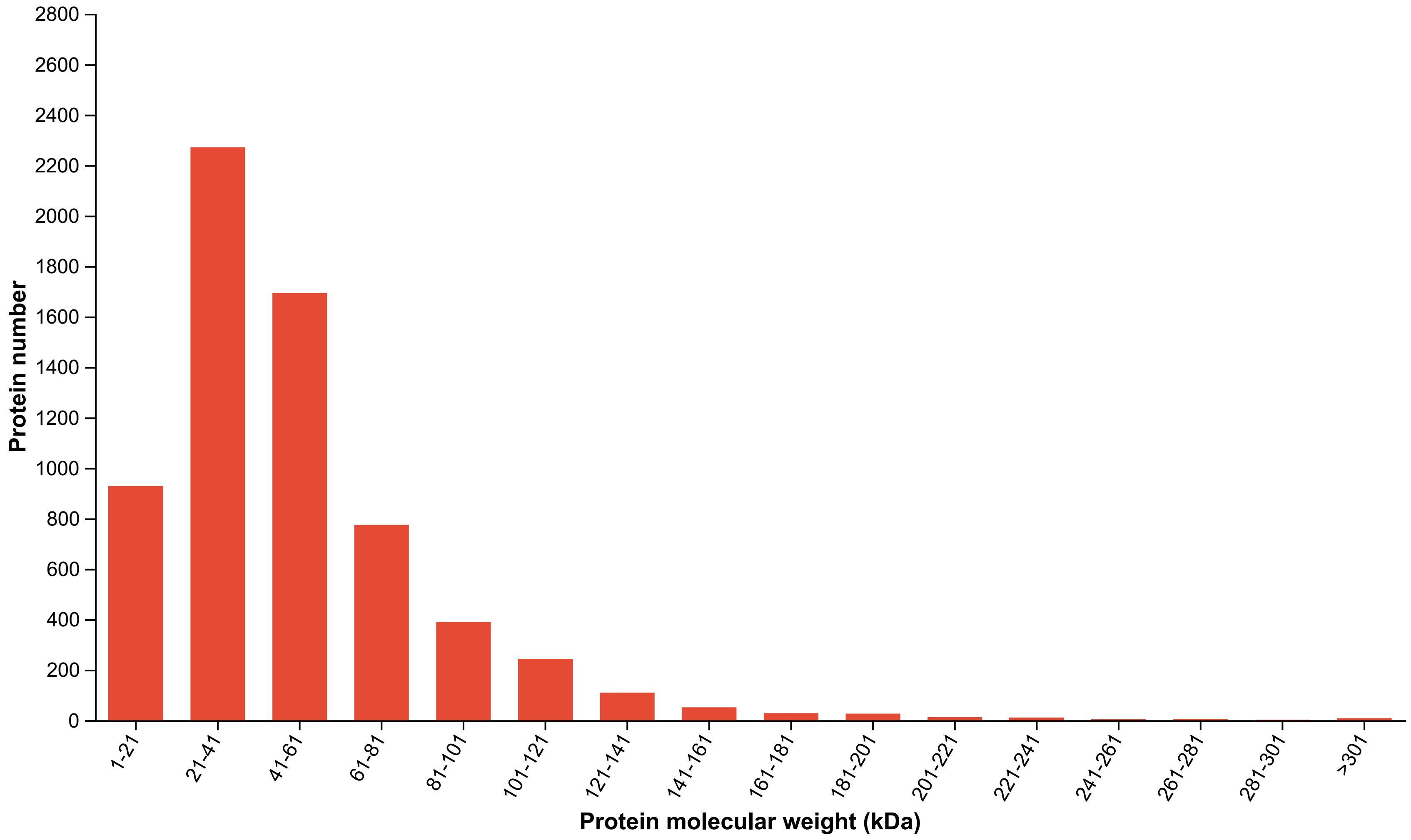

Supplement: Supplementary file 1 [file DataSheet1.zip › Supplementary Material/Figure S1.pdf]
