# Supplementary material for: Combined transcriptomic and proteomic analyses uncover molecular basis of heat tolerance in pakchoi (Brassica rapa subsp. chinensis)
Source: Front Plant Sci. 2026 Mar 11;17:1734608. doi: 10.3389/fpls.2026.1734608 (PMC13014383; doi:10.3389/fpls.2026.1734608)
Supplement: Supplementary file 1 [file DataSheet1.zip › Supplementary Material/Table S1.docx]

**Table S1** RT-qPCR primers

| **Gene name** | **Assay** | **Sequences** |
| --- | --- | --- |
| *BAA01g40860* | RT-qPCR | 5’-GCACCACTCCTTCCTACGTC-3’  5’-ACGCATGAGAGCTGGAATCT-3’ |
| *BAA08g30280* | RT-qPCR | 5’-CAGTCCAGAGCGACATGACT-3’  5’-TCTCCTCGGGAGAAAACTGC-3’ |
| *BAA06g12810* | RT-qPCR | 5’-TGTTCAGAGCGATATGCGGC-3’  5’-TCCTCGGGAGAGAACTGCTT-3’ |
| *BAA05g13130* | RT-qPCR | 5’-TCCCACTTCTTCATTGGGGT-3’  5’-TCGGATTCTCGGAAGCTCAC-3’ |
| *BAA01g16210* | RT-qPCR | 5’-TCCTCTCCGTCAGTGCTAGT-3’  5’-AGAAGGTGAACGCACCTCATC-3’ |
| *BAA02g03270* | RT-qPCR | 5’-ACTGCTGGGTCTAGTTTGGG-3’  5’-ATTGGAGACTTTGGCAGCCT-3’ |
| *BAA01g19180* | RT-qPCR | 5’-AACGAGCGTTAGCTTGCGTA-3’  5’-CTTCCCGGGACGATCATAGC-3’ |
| *BcGAPC* | RT-qPCR | 5’-AGAGCCGCTTCCTTCAACATCATT-3’  5’-TGGGCACACGGAAGGACATACC-3’ |
