# Supplementary material for: Combined transcriptomic and proteomic analyses uncover molecular basis of heat tolerance in pakchoi (Brassica rapa subsp. chinensis)
Source: Front Plant Sci. 2026 Mar 11;17:1734608. doi: 10.3389/fpls.2026.1734608 (PMC13014383; doi:10.3389/fpls.2026.1734608)
Supplement: Supplementary file 1 [file DataSheet1.zip › Supplementary Material/Table S2.docx]

**Table S2** Summary of transcriptome data quality in each sample

| Sample ID | Raw reads | Clean reads | Clean bases | Q20(%) | Q30(%) | GC(%) |
| --- | --- | --- | --- | --- | --- | --- |
| H_R_1 | 43239302 | 42910252 | 6436170611 | 98.69 | 95.80 | 47.89 |
| H_R_2 | 41595340 | 41310974 | 6202937395 | 98.83 | 96.29 | 48.04 |
| H_R_3 | 50610612 | 50295004 | 7546157047 | 98.88 | 96.44 | 48.10 |
| R_1 | 43721024 | 43439662 | 6515144941 | 98.80 | 96.19 | 48.08 |
| R_2 | 40740410 | 40464666 | 6074111685 | 98.81 | 96.21 | 47.51 |
| R_3 | 42479854 | 42190806 | 6334153009 | 98.88 | 96.43 | 47.28 |
