# Supplementary material for: Combined transcriptomic and proteomic analyses uncover molecular basis of heat tolerance in pakchoi (Brassica rapa subsp. chinensis)
Source: Front Plant Sci. 2026 Mar 11;17:1734608. doi: 10.3389/fpls.2026.1734608 (PMC13014383; doi:10.3389/fpls.2026.1734608)
Supplement: Supplementary file 1 [file DataSheet1.zip › Supplementary Material/Table S5.docx]

**Table S5**  Summary of the identified precursors, peptides and proteins in each sample

| Sample ID | Precursors | Peptides | Proteins |
| --- | --- | --- | --- |
| HS-1 | 39,629 | 30,275 | 6,484 |
| HS-2 | 39,610 | 29,603 | 6,466 |
| HS-3 | 39,431 | 29,047 | 6,475 |
| CK-1 | 39,316 | 28,186 | 6,447 |
| CK-2 | 38,805 | 27,360 | 6,452 |
| CK-3 | 39,296 | 27,068 | 6,461 |
